# 1-(4-Chloro­phen­yl)-3-(2-meth­oxy­anilino)propan-1-one

**DOI:** 10.1107/S1600536810054449

**Published:** 2011-01-12

**Authors:** Ligia Llovera, Pavel Anzenbacher, Simón E. López, Teresa González

**Affiliations:** a Laboratorio 223, Departamento de Química, Universidad Simon Bolivar (USB), Apartado 47206, Caracas 1080-A, Venezuela; b Department of Chemistry, Center for Photochemical Sciences, Bowling Green State University (BGSU), Bowling Green, OH 43-403, USA; c Centro de Química, Instituto Venezolano de Investigaciones Científicas, Apartado 21827, Caracas 1020-A, Venezuela

## Abstract

In the title compound, C_16_H_16_ClNO_2_, the mol­ecule adopts a bowed conformation, with a dihedral angle of 39.9 (2)° between the aromatic rings. In the crystal, mol­ecules are linked by C—H⋯O hydrogen bonds, generating *C*(6) chains propagating in [010]. Very weak aromatic π–π stacking is also observed [centroid–centroid distance = 4.040 (2) Å].

## Related literature

For the synthesis of quinoline derivatives, see: Peifer *et al.* (2007 ▶). For background to the anti­microbial activity of quinolines, see: Yamashkin & Oreshkina (2006 ▶). For further synthetic details, see: Dienys *et al.* (1977 ▶); Volkov *et al.* (2007 ▶).
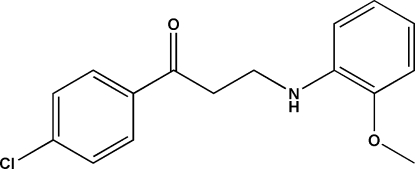


## Experimental

### 

#### Crystal data

C_16_H_16_ClNO_2_
*M* *_r_* = 289.75
Orthorhombic, 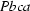

*a* = 7.1690 (6) Å
*b* = 14.4303 (11) Å
*c* = 28.667 (3) Å
*V* = 2965.6 (4) Å^3^
*Z* = 8
Mo *K*α radiation
μ = 0.26 mm^−1^
*T* = 293 K
0.48 × 0.36 × 0.20 mm

#### Data collection

Rigaku AFC-7S Mercury diffractometer
Absorption correction: multi-scan (*REQAB*; Jacobson, 1998 ▶) *T*_min_ = 0.927, *T*_max_ = 0.950
31012 measured reflections
3035 independent reflections
2016 reflections with *I* > 2σ(*I*)
*R*_int_ = 0.057

#### Refinement

*R*[*F* ^2^ > 2σ(*F* ^2^)] = 0.077
*wR*(*F* ^2^) = 0.198
*S* = 1.14
3035 reflections
182 parameters
H-atom parameters constrained
Δρ_max_ = 0.16 e Å^−3^
Δρ_min_ = −0.26 e Å^−3^

### 

Data collection: *CrystalClear* (Rigaku/MSC, 2005 ▶); cell refinement: *CrystalClear*; data reduction: *CrystalClear*; program(s) used to solve structure: *CrystalStructure* (Rigaku/MSC, 2005) ▶ and *SHELXTL* (Sheldrick, 2008 ▶); program(s) used to refine structure: *SHELXTL*; molecular graphics: *SHELXTL* and *DIAMOND* (Brandenburg, 1999 ▶); software used to prepare material for publication: *SHELXTL* and *PLATON* (Spek, 2009 ▶).

## Supplementary Material

Crystal structure: contains datablocks global, I. DOI: 10.1107/S1600536810054449/hb5773sup1.cif

Structure factors: contains datablocks I. DOI: 10.1107/S1600536810054449/hb5773Isup2.hkl

Additional supplementary materials:  crystallographic information; 3D view; checkCIF report

## Figures and Tables

**Table 1:** Hydrogen-bond geometry (Å, °)

*D*—H⋯*A*	*D*—H	H⋯*A*	*D*⋯*A*	*D*—H⋯*A*
C15—H15*A*⋯O2^i^	0.93	2.49	3.414 (4)	171

Symmetry code: (i) 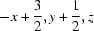.

**Fig. 1.**
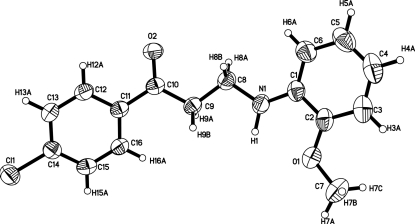
The molecular structure of (I), showing displacement elipsoids drawn at the 35% probability level and H atoms shown as spheres of arbitrary radii.

**Fig. 2.**
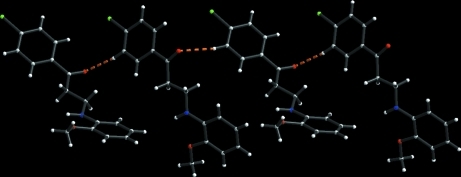
View of infinite chains interconnected through hydrogen bonding interactions of the kind C—H···O along the bc plane. Dashed lines indicate the donor···acceptor interactions for hydrogen bonds.

**Fig. 3.**
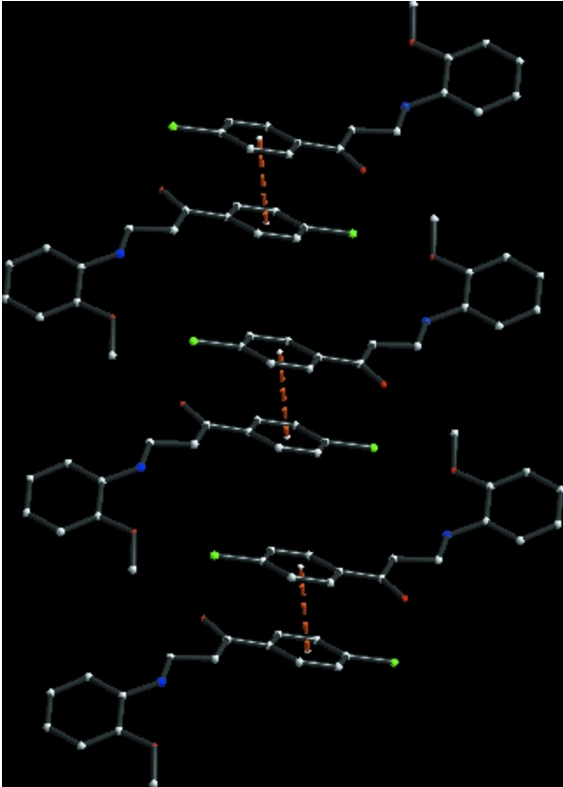
View of the weak interactions of the kind π···π in the structure

## supplementary crystallographic information

### Comment

The title compound was prepared as an intermediate for the synthesis of 4-aryl-8-methoxy-quinoline under acid conditions (Dienys *et al.*, 1977). The synthesis of the title compound might be obtained through decyclization of piperidol and transamination of the decyclization products (Volkov *et al.*, 2007). These compounds exhibit a broad range of antimicrobial activity and particular, antitubercular activity, antimalarial activity and are also present in antiallergic and antiasthmatic agents (Yamashkin & Oreshkina, 2006). In addition, these compounds could act as drug targets of a large numbers of protein-inhibitor complexes, for example the mitogen-activated protein kinase (Peifer *et al.*, 2007).

The X-ray structure determination showed that compound (I) contains only one organic molecule per asymmetric unit (Fig. 1). The molecule adopts a slightly angular conformation, where the dihedral angle defined by aromatic rings is 39.9 (2)°. respectively. The crystal packing (Fig. 2) of this structure consists of infinite chains which are interconnected through hydrogen bonding interactions of the kind C—H···O (3.415 Å) along the *bc* plane. The final array (Fig. 3) is sustained by weak interactions of the kind π···π between aromatics rings with distance between centroid to centroid, *Cg*2···*Cg*2: 4.040 (2) Å. Where *Cg*2 is defined by C11/C12/C13/C14/C15/C16 atoms.

### Experimental

A solution of 3-(4-chlorophenyl)-*N*,*N*-dimethyl-3-oxopropan-1-aminium chloride (0.01 mol) in distilled water (5 ml) was stirred at room temperature in a round bottom flask. After 5 minutes, a solution of 2-methoxy-phenylamine (0.01 mol) and concentrated hydrochloric acid (0.5 ml) in ethanol (10 ml) was added dropwise and the mixture was stirred at room temperature for 12 h to yield yellow blocks of (I).  Yield: 79%. *M*.p. 83–84°C; ^1^H NMR (400 MHz, CDCl_3_, δ (p.p.m.), J= Hz): 3.27 (t, 2H, J= 6.4), 3.64 (t, 2H, J= 6.4), 3.81 (s, 3H), 4.57 (s, 1H), 6.68 (m, 2H), 6.76 (dd, 1H, J= 8.4, 1.5), 6.88 (td, 1H, J= 7.6, 1.1), 7.42 (d, 2H, J= 8.4), 7.87 (d, 2H, J= 8.4). ^13^C NMR (100 MHz, CDCl_3_, δ (p.p.m.)): 38.0 (C9), 38.4 (C8), 55.5 (C7), 109.7 (C3), 109.9 (C6), 116.9 (C4), 121.3 (C5), 129.0 (C13 and C15), 129.5 (C12 and C16), 135.2 (C1), 137.6 (C11), 139.8 (C14), 147.2 (C7), 200.0 (C10). IR (KBr, cm^-1^): 3413, 3085, 3061, 2961, 1685, 1074, 792. EI—MS (m/*z*): 290.37 [*M*^+&bull;^], 292.37 [*M*^+&bull;^ +2], 136.07 [*M*^+&bull;^ – (4-ClPhCOCH_2_)].

### Refinement

The N-bound H atoms were located in difference maps and refined as riding in their as found relative positions with *U*_iso_(H) = 1.5*U*_eq_(N). The C-bound H atoms were placed in idealized positions (C—H = 0.93–0.98 Å) and refined as riding with *U*_iso_(H) = 1.2*U*_eq_(C).

### Crystal data

**Table:**

C_16_H_16_ClNO_2_	*F*(000) = 1216
*M**_r_* = 289.75	*D*_x_ = 1.298 Mg m^−^^3^
Orthorhombic, *P**b**c**a*	Mo *K*α radiation, λ = 0.71070 Å
Hall symbol: -P 2ac 2ab	Cell parameters from 13752 reflections
*a* = 7.1690 (6) Å	θ = 2.8–56.1°
*b* = 14.4303 (11) Å	µ = 0.26 mm^−^^1^
*c* = 28.667 (3) Å	*T* = 293 K
*V* = 2965.6 (4) Å^3^	Block, yellow
*Z* = 8	0.48 × 0.36 × 0.20 mm

### Data collection

**Table:**

Rigaku AFC-7S Mercury diffractometer	3035 independent reflections
Radiation source: fine-focus sealed tube	2016 reflections with *I* > 2σ(*I*)
graphite	*R*_int_ = 0.057
ω scans	θ_max_ = 28.0°, θ_min_ = 2.8°
Absorption correction: multi-scan (REQAB; Jacobson, 1998)	*h* = −8→8
*T*_min_ = 0.927, *T*_max_ = 0.950	*k* = −17→13
31012 measured reflections	*l* = −34→34

### Refinement

**Table:**

Refinement on *F*^2^	Secondary atom site location: difference Fourier map
Least-squares matrix: full	Hydrogen site location: inferred from neighbouring sites
*R*[*F*^2^ > 2σ(*F*^2^)] = 0.077	H-atom parameters constrained
*wR*(*F*^2^) = 0.198	*w* = 1/[σ^2^(*F*_o_^2^) + (0.0693*P*)^2^ + 1.923*P*] where *P* = (*F*_o_^2^ + 2*F*_c_^2^)/3
*S* = 1.14	(Δ/σ)_max_ < 0.001
3035 reflections	Δρ_max_ = 0.16 e Å^−^^3^
182 parameters	Δρ_min_ = −0.26 e Å^−^^3^
0 restraints	Extinction correction: *SHELXTL* (Sheldrick, 2008), Fc^*^=kFc[1+0.001xFc^2^λ^3^/sin(2θ)]^-1/4^
Primary atom site location: structure-invariant direct methods	Extinction coefficient: 0.0045 (11)

### Special details

**Table:**

Geometry. All e.s.d.'s (except the e.s.d. in the dihedral angle between two l.s. planes) are estimated using the full covariance matrix. The cell e.s.d.'s are taken into account individually in the estimation of e.s.d.'s in distances, angles and torsion angles; correlations between e.s.d.'s in cell parameters are only used when they are defined by crystal symmetry. An approximate (isotropic) treatment of cell e.s.d.'s is used for estimating e.s.d.'s involving l.s. planes.
Refinement. Refinement of *F*^2^ against ALL reflections. The weighted *R*-factor *wR* and goodness of fit *S* are based on *F*^2^, conventional *R*-factors *R* are based on *F*, with *F* set to zero for negative *F*^2^. The threshold expression of *F*^2^ > σ(*F*^2^) is used only for calculating *R*-factors(gt) *etc*. and is not relevant to the choice of reflections for refinement. *R*-factors based on *F*^2^ are statistically about twice as large as those based on *F*, and *R*- factors based on ALL data will be even larger.

### Fractional atomic coordinates and isotropic or equivalent isotropic displacement parameters (Å^2^)

**Table:**

	*x*	*y*	*z*	*U*_iso_*/*U*_eq_	
Cl1	0.69107 (15)	0.62967 (7)	0.61228 (4)	0.0854 (4)	
O1	0.4661 (4)	0.2337 (2)	0.30790 (9)	0.0883 (9)	
O2	0.9890 (4)	0.25566 (16)	0.49786 (9)	0.0727 (7)	
N1	0.7477 (5)	0.2272 (2)	0.36535 (10)	0.0684 (8)	
H1	0.6749	0.2852	0.3609	0.103*	
C1	0.7033 (5)	0.1459 (2)	0.34171 (11)	0.0623 (9)	
C2	0.5513 (6)	0.1488 (3)	0.31054 (12)	0.0690 (10)	
C3	0.5005 (7)	0.0718 (3)	0.28559 (14)	0.0866 (13)	
H3A	0.4010	0.0746	0.2648	0.104*	
C4	0.5979 (9)	−0.0100 (3)	0.29143 (16)	0.1005 (16)	
H4A	0.5628	−0.0626	0.2748	0.121*	
C5	0.7461 (9)	−0.0141 (3)	0.32167 (16)	0.0958 (15)	
H5A	0.8109	−0.0695	0.3254	0.115*	
C6	0.8003 (6)	0.0638 (3)	0.34675 (13)	0.0783 (11)	
H6A	0.9018	0.0607	0.3669	0.094*	
C7	0.3001 (8)	0.2415 (4)	0.28063 (18)	0.123 (2)	
H7A	0.2579	0.3046	0.2809	0.185*	
H7B	0.2051	0.2023	0.2935	0.185*	
H7C	0.3257	0.2229	0.2491	0.185*	
C8	0.8471 (5)	0.2253 (2)	0.40935 (12)	0.0654 (9)	
H8A	0.9791	0.2153	0.4038	0.078*	
H8B	0.8010	0.1748	0.4285	0.078*	
C9	0.8179 (5)	0.3165 (2)	0.43408 (11)	0.0573 (8)	
H9A	0.8793	0.3651	0.4164	0.069*	
H9B	0.6855	0.3304	0.4346	0.069*	
C10	0.8898 (5)	0.3180 (2)	0.48287 (11)	0.0554 (8)	
C11	0.8363 (4)	0.3968 (2)	0.51434 (11)	0.0541 (8)	
C12	0.8721 (5)	0.3894 (2)	0.56181 (12)	0.0667 (10)	
H12A	0.9276	0.3360	0.5735	0.080*	
C13	0.8258 (5)	0.4607 (3)	0.59179 (12)	0.0697 (10)	
H13A	0.8488	0.4551	0.6236	0.084*	
C14	0.7456 (5)	0.5401 (2)	0.57432 (12)	0.0605 (9)	
C15	0.7108 (5)	0.5491 (2)	0.52753 (13)	0.0621 (9)	
H15A	0.6576	0.6032	0.5159	0.074*	
C16	0.7554 (5)	0.4773 (2)	0.49781 (12)	0.0580 (8)	
H16A	0.7306	0.4831	0.4661	0.070*	

### Atomic displacement parameters (Å^2^)

**Table:**

	*U*^11^	*U*^22^	*U*^33^	*U*^12^	*U*^13^	*U*^23^
Cl1	0.0803 (8)	0.0852 (7)	0.0905 (8)	0.0111 (5)	−0.0050 (5)	−0.0216 (5)
O1	0.093 (2)	0.092 (2)	0.0794 (19)	0.0084 (16)	−0.0267 (15)	−0.0150 (14)
O2	0.0761 (17)	0.0629 (15)	0.0790 (16)	0.0129 (12)	−0.0181 (13)	0.0009 (12)
N1	0.086 (2)	0.0609 (17)	0.0581 (17)	−0.0038 (15)	−0.0151 (16)	0.0005 (13)
C1	0.076 (2)	0.062 (2)	0.0493 (19)	−0.0098 (18)	0.0071 (17)	0.0004 (15)
C2	0.085 (3)	0.071 (2)	0.051 (2)	−0.013 (2)	0.0041 (19)	−0.0045 (17)
C3	0.104 (3)	0.089 (3)	0.067 (3)	−0.026 (3)	0.005 (2)	−0.011 (2)
C4	0.148 (5)	0.080 (3)	0.074 (3)	−0.035 (3)	0.017 (3)	−0.016 (2)
C5	0.148 (5)	0.058 (2)	0.081 (3)	0.001 (3)	0.024 (3)	0.002 (2)
C6	0.100 (3)	0.065 (2)	0.070 (2)	−0.001 (2)	0.008 (2)	0.0031 (19)
C7	0.114 (4)	0.147 (5)	0.109 (4)	0.022 (3)	−0.050 (3)	−0.021 (3)
C8	0.065 (2)	0.066 (2)	0.064 (2)	−0.0021 (17)	−0.0099 (18)	−0.0009 (16)
C9	0.0517 (19)	0.061 (2)	0.059 (2)	−0.0049 (15)	−0.0056 (15)	0.0038 (15)
C10	0.0472 (18)	0.0536 (19)	0.065 (2)	−0.0073 (15)	−0.0069 (15)	0.0057 (15)
C11	0.0432 (17)	0.0570 (19)	0.062 (2)	−0.0058 (14)	−0.0074 (15)	0.0039 (15)
C12	0.073 (2)	0.062 (2)	0.065 (2)	0.0093 (17)	−0.0146 (18)	0.0044 (16)
C13	0.077 (2)	0.078 (2)	0.054 (2)	0.0080 (19)	−0.0122 (18)	−0.0015 (18)
C14	0.0523 (19)	0.061 (2)	0.068 (2)	−0.0029 (16)	−0.0025 (17)	−0.0043 (16)
C15	0.056 (2)	0.0540 (19)	0.076 (2)	−0.0010 (15)	−0.0092 (17)	0.0093 (17)
C16	0.0564 (19)	0.058 (2)	0.060 (2)	−0.0017 (15)	−0.0091 (16)	0.0050 (15)

### Geometric parameters (Å, °)

**Table:**

Cl1—C14	1.734 (3)	C7—H7C	0.9600
O1—C2	1.371 (4)	C8—C9	1.510 (4)
O1—C7	1.428 (5)	C8—H8A	0.9700
O2—C10	1.224 (4)	C8—H8B	0.9700
N1—C1	1.392 (4)	C9—C10	1.491 (4)
N1—C8	1.449 (4)	C9—H9A	0.9700
N1—H1	0.9952	C9—H9B	0.9700
C1—C6	1.381 (5)	C10—C11	1.501 (5)
C1—C2	1.410 (5)	C11—C16	1.382 (4)
C2—C3	1.370 (5)	C11—C12	1.389 (5)
C3—C4	1.382 (7)	C12—C13	1.381 (5)
C3—H3A	0.9300	C12—H12A	0.9300
C4—C5	1.373 (7)	C13—C14	1.377 (5)
C4—H4A	0.9300	C13—H13A	0.9300
C5—C6	1.390 (6)	C14—C15	1.371 (5)
C5—H5A	0.9300	C15—C16	1.379 (5)
C6—H6A	0.9300	C15—H15A	0.9300
C7—H7A	0.9600	C16—H16A	0.9300
C7—H7B	0.9600		
			
C2—O1—C7	118.2 (3)	N1—C8—H8B	109.9
C1—N1—C8	121.3 (3)	C9—C8—H8B	109.9
C1—N1—H1	121.8	H8A—C8—H8B	108.3
C8—N1—H1	112.7	C10—C9—C8	113.9 (3)
C6—C1—N1	123.8 (3)	C10—C9—H9A	108.8
C6—C1—C2	118.8 (3)	C8—C9—H9A	108.8
N1—C1—C2	117.4 (3)	C10—C9—H9B	108.8
C3—C2—O1	125.3 (4)	C8—C9—H9B	108.8
C3—C2—C1	120.8 (4)	H9A—C9—H9B	107.7
O1—C2—C1	113.9 (3)	O2—C10—C9	121.3 (3)
C2—C3—C4	119.7 (4)	O2—C10—C11	119.6 (3)
C2—C3—H3A	120.1	C9—C10—C11	119.1 (3)
C4—C3—H3A	120.1	C16—C11—C12	118.6 (3)
C5—C4—C3	120.3 (4)	C16—C11—C10	122.5 (3)
C5—C4—H4A	119.9	C12—C11—C10	118.9 (3)
C3—C4—H4A	119.9	C13—C12—C11	120.5 (3)
C4—C5—C6	120.6 (4)	C13—C12—H12A	119.7
C4—C5—H5A	119.7	C11—C12—H12A	119.7
C6—C5—H5A	119.7	C14—C13—C12	119.6 (3)
C1—C6—C5	119.9 (4)	C14—C13—H13A	120.2
C1—C6—H6A	120.0	C12—C13—H13A	120.2
C5—C6—H6A	120.0	C15—C14—C13	120.8 (3)
O1—C7—H7A	109.5	C15—C14—Cl1	120.1 (3)
O1—C7—H7B	109.5	C13—C14—Cl1	119.1 (3)
H7A—C7—H7B	109.5	C14—C15—C16	119.4 (3)
O1—C7—H7C	109.5	C14—C15—H15A	120.3
H7A—C7—H7C	109.5	C16—C15—H15A	120.3
H7B—C7—H7C	109.5	C15—C16—C11	121.1 (3)
N1—C8—C9	108.9 (3)	C15—C16—H16A	119.4
N1—C8—H8A	109.9	C11—C16—H16A	119.4
C9—C8—H8A	109.9		

### Hydrogen-bond geometry (Å, °)

**Table:**

*D*—H···*A*	*D*—H	H···*A*	*D*···*A*	*D*—H···*A*
C15—H15A···O2^i^	0.93	2.49	3.414 (4)	171

Symmetry codes: (i) −*x*+3/2, *y*+1/2, *z*.
